# Bone Targeted Therapies for Bone Metastasis in Breast Cancer

**DOI:** 10.3390/jcm2040176

**Published:** 2013-10-14

**Authors:** Wajeeha Razaq

**Affiliations:** Stephenson Cancer Center, The University of Oklahoma, Norman, OK 73104, USA; E-Mail: wajeeha-razaq@ouhsc.edu; Tel.: +405-271-4022; Fax: +405-271-4221

**Keywords:** bone metastasis, bisphosphonates, bone-targeted therapy, radiopharmaceuticals

## Abstract

Cancer metastasis to the bone develops commonly in patients with various malignancies, and is a major cause of morbidity and diminished quality of life in many affected patients. Emerging treatments for metastatic bone disease have arisen from advances in our understanding of the unique cellular and molecular mechanisms that contribute to the bone metastasis. The tendency of cancer cells to metastasize to bone is probably the end result of many factors including vascular pathways, the highly vascular nature of the bone marrow (which increases the probability that cancer cells will be deposited in bone marrow capillaries), and molecular characteristics of the cancer cells that allow them to adapt to the bone marrow microenvironment. The goals of treating osseous metastases are manifold. Proper treatment can lead to significant improvements in pain control and function, and maintain skeletal integrity. The treatment plan requires a multidisciplinary approach. Widespread metastatic disease necessitates systemic therapy, while a localized problem is best managed with surgery, external beam radiotherapy, or both. Patients with bone metastasis can have prolonged survival, and proper management can have a significant impact on their quality of life. We will review the factors in this article that are promising molecular bone-targeted therapies or will be likely targets for future therapeutic intervention to restore bone remodeling and suppress tumor growth.

## 1. Introduction

Cancer metastasis to the bone develops commonly in patients with various malignancies, and is a major cause of morbidity and diminished quality of life in many affected patients. The effective treatment of bone metastasis requires a multidisciplinary approach by medical, surgical and radiation oncologists. Emerging treatments for metastatic bone disease have arisen from advances in our understanding of the unique cellular and molecular mechanisms that contribute to the bone metastasis. The tendency of cancer cells to metastasize to bone is probably the end result of many factors including vascular pathways, the highly vascular nature of the bone marrow (which increases the probability that cancer cells will be deposited in bone marrow capillaries), and molecular characteristics of the cancer cells that allow them to adapt to the bone marrow microenvironment [1,2]. In fact, breast cancer cells have been shown to adopt an osteoblast-like phenotype that may help them survive in the bone marrow [3]. Once in the bone marrow cancer stem cells may remain dormant for prolonged periods of time; however, the specific signals that control cancer stem cells dormancy and reactivation are currently not well understood [1].

This metastasis can result in substantial morbidity in the form of skeletal related events that are defined as pathological fractures, spinal cord compression, hypercalcemia or pain requiring radiation or surgery of the bone. Once bone metastases are diagnosed, they are usually incurable and the goal of treatment becomes focused on palliation and prevention of skeletal related events. Treatment modalities for bone metastasis include chemotherapy, hormonal therapy, analgesics, radiotherapy, and orthopedic surgery. In addition, bisphosphonates are potent antiresorptives, used in the prevention of skeletal related events, but they are not completely effective in inhibiting the progression of bone metastasis. A new strategy is bone-targeted therapy. In this review, bone-targeted treatment relating to breast cancer metastasis will be discussed.

## 2. Mechanism of Bone Metastasis

Breast cancer cells in the bone marrow alter the functions of bone-resorbing (osteoclasts) and bone-forming cells (osteoblasts) and thereby disrupting physiological bone remodeling [4,5]. Breast cancer cells may cause stimulation of osteoclast differentiation and maturation along with secreting factors that inhibit osteoblast differentiation and activity. Their interaction with osteoblasts also induces the release cytokines that promote tumor growth [4,5]. This leads to an imbalance between bone resorption and formation, resulting in enhanced skeletal destruction and as a consequence of osteolysis, occurrence of pathological fractures. 

Several molecules that are produced by breast cancer—e.g., parathyroid hormone-related protein, interleukins (IL-6, IL-8 and IL-11), cytokines (macrophage stimulating factor (M_CSF)) and prostaglandins—stimulate osteoclast active through the activation of the receptor activator of nuclear factor kB ligand (RANKL)/RANK pathway, which is the primary mediator of osteoclast-mediated bone resorption [5,6]. Breast cancer cells also secrete activin A (a member of transforming growth factor (TGF)-β), noggin (a bone morphogenetic protein (BMP) antagonist) and dikkopf-1 (DKK-1; a wingless (Wnt) protein antagonist), all of them inhibiting osteoblast differentiation [5,7]. As bone is resorbed, growth factors (like TGF-β and insulin-like growth factor-I) stored in the bone matrix are then released and stimulate breast cancer cell proliferation, providing a supportive niche for tumor growth [5,6,7]. 

The goals of treating osseous metastases are manifold. Proper treatment can lead to significant improvements in pain control and function, and maintain skeletal integrity. The treatment plan requires a multidisciplinary approach. Widespread metastatic disease necessitates systemic therapy, while a localized problem is best managed with surgery, external beam radiotherapy, or both. Patients with bone metastasis can have prolonged survival, and proper management can have a significant impact on their quality of life. 

### 2.1. Bisphosphonates

The realization that normal cells in the bone microenvironment support the development of skeletal lesions has led to the use of bisphosphonates, as inhibitors of osteoclast-mediated bone resorption, in the treatment of patients with bone metastasis [4]. Nitrogen-containing bisphosphonates (N-BP) specifically inhibit osteoclast farnesyl pyrophosphate synthase activity, a key enzyme in the mevalonate pathway, which causes the inhibition of prenyllation of small GTPases and the subsequent inactivation of osteoclasts. In addition to inhibition of osteoclast activation and function, a growing body of preclinical data suggests that N-BPs exert direct and/or indirect antitumor effects. There is abundant evidence of the inherent antitumor activity of N-BPs *in vitro*, including induction of tumor cell apoptosis, inhibition of tumor cell proliferation, migration, and invasion [8]. *In vitro*, N-BPs (zoledronate, risedronate, alendronate, ibandronate) also interfere with all major steps of the angiogenic process, such as endothelial cell migration, proliferation and tube formation [8]. Despite these theoretical advantages, the data are inconsistent on what impact bisphosphonates have on breast cancer outcomes. In a meta-analysis of trials that randomized patients to bisphosphonates or not in the adjuvant setting failed to find significant reductions in the overall number of deaths, bone metastases, overall disease recurrences, distant relapse, visceral recurrences, or local relapses [9]. Nevertheless, in subgroup analysis, use of zoledronic acid was associated with a statistically significant lower risk for disease recurrence but not death or bone metastases rates. 

However, the role of osteoclast inhibitors in preventing breast cancer recurrence and improving outcomes continues to be an active subject of clinical research. 

We will review the factors in this article that are promising molecular bone-targeted therapies or will be likely targets for future therapeutic intervention to restore bone remodeling and suppress tumor growth.

### 2.2. Osteoclast—Mediated Bone Resorption Inhibition

RANK/RANKL (osteoclast formation), integrin, c-Src, and cathepsin K (osteoclast function), all represent suitable targets for inhibition of pathological bone resorption.

#### 2.2.1. RANK/RANKL

RANKL is a transmembrane protein expressed on the surface of osteoblasts that can be cleaved as a soluble form by proteases. Both the membrane-bound and soluble forms of RANKL attach to RANK, a receptor on the cell surface of osteolclast precursors, to stimulate osteoclastogenesis [6]. On the other hand, osteoblasts secrete RANKL inhibitor that inhibits RANKL/RANK interaction. The balance between the RANKL and RANKL inhibitor regulates the process of bone resorption and the imbalance of this process has been seen in many cancers, including breast cancer [5].

Denosumab is a fully human monoclonal antibody that specifically targets RANKL. This antibody is currently a very promising potential alternative to bisphosphonates. Several studies have compared the efficacy of denosumab and bisphosphonates in patients with breast cancer, with promising results. A randomized phase II, open label trial, examined sequential osteoclast inhibiting therapy on the biochemical marker of bone resorption, urinary N-telopeptide (uNTx) [10]. One hundred and eleven patients with metastatic bone disease from a variety of tumors including breast with uNTx levels above 50 nmol/L were randomized to either continuing same IV bisphosphonates or switching to denosumab therapy. A significantly greater percentage of patients on denosumab *vs*. IV bisphosphonates reduced the uNTx below 50 nmol/L [10]. Another study examined non-inferiority as the primary endpoint when compared to zoledronic acid, and superiority as a secondary endpoint [11]. Denosumab met both of these endpoints with denosumab showing a greater delay to first on study SREs than zoledronic acid [11]. Denosumab has been well tolerated in these trials. The risk of osteonecrosis of the jaw was not significantly different between denosumab and zolendronic acid, but the renal toxicity was less frequent with denosumab [11]. Although increased risk of infection and secondary neoplasms with densosumab is a potential concern, since RANKL also regulates immune function involving dendritic cells and T and B cells [12]. Long term follow up is needed to clarify this risk. 

#### 2.2.2. Cathepsin K

Cathepsin K is a lysomal cysteine protease highly expressed in osteoclasts that plays a major role in bone resorption [5,13]. The function of cathepsin K in osteoclasts was first revealed by the finding of a loss of function mutation in the human cathepsin K gene in patients with pycnodysostosic, a rare genetic disorder characterized by impaired osteoclastic bone resorption [13]. Also it was noticed that pycnodysostosis phenotype can be reproduced in mice if cathepsin K was genetically impaired. Overall, these findings indicate that cathepsin K plays a key role in osteoclast-mediated bone resorption. After this discovery, it was postulated that molecules that inhibit cathepsin K activity could serve as useful therapeutic agents against diseases associated with excessive levels of bone destruction. Moreover, it was noticed that cathepsin K inhibitors not only inhibit bone resorption but also stimulate bone formation [14]. This way it seems cathepsin K inhibitors have an advantage over other antiresorptive agents in the treatment of diseases associated with bone loss. 

A number of cathepsin K inhibitors have been examined in animal models of estrogen deficiency osteoporosis. Development of two of these compounds relacatib and balicatib, was stopped because of side effects thought to be due to lack of specificity [15]. An increased incidence of skin rashes and morphea-like skin adverse events has been reported in a phase 2b study of balicatib [16,17]. Three cathepsin K inhibitors are currently in development for treatment of osteoporosis. Odanacatib is in phase 3, ONO-5334 is in phase 2, and MIV-711 is in preclinical development. Thus, direct inhibition of cathepsin K, the enzyme primarily responsible for degradation of bone matrix by osteoclasts, shows promise for treatment of postmenopausal osteoporosis and perhaps other bone diseases. Further development in oncology field is still on hold. 

#### 2.2.3. c-Src

c-Src, a member of the non-receptor tyrosine kinase family, has role in a number of signaling pathways that regulate malignant cell proliferation, angiogenesis, adhesion, invasion, motility, survival and metastasis [18,19,20]. It is overexpressed in breast cancer tissue as well as in colon, lung and skin [21]. It positively regulates osteoclasts and negatively regulates osteoblasts via complex signaling pathways [22,23]. It has been seen that targeted disruption of c-Src in mice resulted in development of osteoperosis characterized by thick bone trabeculae secondary to osteoclast dysfunction, suggesting a role for c-Src in bone remodeling [24]. c-Src activity is associated with the capability of breast cancer cells to metastasize to bone. A study showed that a Src gene-expression signature was associated with late onset of bone metastasis in breast cancer, independent of estrogen receptor status or breast cancer subtype [25]. Preclinical studies showed that c-Src inhibiors decrease osteoclastic bone resorption [26]. Therefore, c-Src is a promising target for suppressing tumor-induced osteolysis and tumor growth.

Currently, three c-Src inhibitors are in the breast cancer trials: dasatinib, bosutinib and saracatinib. Dasatinib is an oral tyrosine kinase inhibitor of Src family kinases and act at Philadelphia chromosome BCR-ABL [27]. Dasatinib is approved for imatinib-intolerant or resistant chronic myeloid leukemia CML and Philadelphia chromosome-positive acute lymphoblastic leukemia. A phase II study in patients with locally advanced or metastatic triple negative breast cancer with dasatinib (*n*-44) was completed. Although the results were not very impressive, it was suggested that selected patients may benefit from Src inhibitors [28]. Several phase II studies are ongoing using dasatinib as a single agent or combined with zoledronic acid in breast cancer with bone metastasis, and combined with either aromatase inhibitors or chemotherapeutic agents in metastatic breast cancer. 

Saracatinib, a dual inhibitor of Src/Abl, has been shown to decrease levels of bone resorption markers in a phase I study in patients with solid tumors [29]. Phase II studies are ongoing to evaluate saracatinib effects in patients with breast cancer and bone metastases. One phase II study is comparing the effects of saracatinib and zoledronic acid in patients with breast and prostate cancers with bone metastasis. 

Bosutinib is also an oral tyrosine kinase inhibitor of both Src family and ABL. Bosutinib inhibits Src-mediated signaling pathways involved in breast cancer cell proliferation, angiogenesis, growth factor expression, motility, and invasion [30]. It is being assessed in phase III trials for CML and Philadelphia positive acute lymphoblastic leukemia. A phase II study of single agent bosutinib in metastatic breast cancer is under way. The combinations with aromatase inhibitors or chemotherapeutic agents with bosutinib are also being evaluated in metastatic breast cancer patients.

#### 2.2.4. Integrins

Integrins are a family of cell surface receptors that primarily mediate interactions of cells with components of the extracellular matrix. Although osteoclasts express various integrins, it is generally well accepted now that avb3 integrin is a central molecule for osteoclast function [31]. There are several ongoing clinical trials evaluating the anticancer effect integrin antagonists in advanced refractory and metastatic cancers [32,33]. ATN-161, IMGN388 and L-000845704 are being evaluated in phase I/II studies and it would be interesting to examine the effects of these agents in clinical oncology [32,33]. 

## 3. Novel Targets from the Bone Microenvironment

### 3.1. CXCL-12/CXCR-4

C-X-C chemokine receptor type-4 (CXCR-4) is a chemokine receptor for stromal cell derived factor-1 (SDF-1 or chemokine ligand-12 CXCL-12). Several types of cancer over express CXCR-4 including breast cancer tissue but the normal breast tissue has low expression [34]. Also CXCR-4 is much more highly expressed in bone metastasis than in visceral metastasis [35]. The CXCR-4/SDF-1 axis is an attractive therapeutic target because of its role in bone metastasis. 

CTCE-9908 is a synthetic peptidic antagonist that reduces the formation of experimental lung and bone metastasis caused by CXCR-4 expressing breast cancer cells [36,37]. In a mouse model, treatment with CTCE-9908 did not reduce the frequency of metastasis, but did decrease both the tumor burden of breast cancer in bone, other organs, and also of the primary breast tumor [36]. A phase I/II study using CCE-9908 was conducted in 25 patients with refractory solid cancers. Tolerance was good and response was modest with progression of disease in 17 patients and stable disease in 5 patients [38]. The short half-life of CTCE-9908 may be a barrier to its use in breast cancer. AMD3100 (PLERIXAFOR) is a CXCR-4 inhibitor that promotes hematopoietic stem cells to mobilize from the bone marrow into the blood stream and is approved for use in autologous transplantation [39]. Clinical trials with this agent alone or with combination of bisphosphonates are also underway. 

### 3.2. Transforming Growth Factor β (TGF-β)

TGF-β binds to a heteromeric complex of transmembrane serine/threonine kinases, the type I and type II receptors, activin receptor-like kinase 5 (ALK5) and transforming growth factor-beta receptor type II TβRII, which phosphorylate and activate the TGF-β specific intracellular signaling mediators Smad2 and Smad3. The phosphorylated Smad2/3 complex then binds Smad4 and translocates to the nucleus, where it regulates the transcription of TGF-β target genes [40]. It regulates the expression of many factors (integrin, IL-6, IL-11, matrix metalloproteinase-1 (MMP-1), CXCR-4) that are involved in bone metastasis formation [40]. Thus, the blockade of TGF-β signaling offers a target for therapeutic intervention to decrease bone metastasis. So far there are no TGF-related drugs in clinical trials for breast cancer with bone metastasis, although TGF inhibitors have been investigated in other types of solid cancers. AP12009 (Trabedersen), a TGF-β2-specific antisense oligonucleotide, showed encouraging results in patients with stage III/IV pancreatic cancer, colorectal cancer and malignant melanoma in a phase I/II study [41]. The SAPPHIRE study (Efficacy and Safety of AP12009 in Adult Patients with Recurrent or Refractory Anaplastic Astrocytoma (WHO grade III) as Compared to Standard Treatment with Temozolomide or Carmustine: A Randomized, Actively Controlled, Open-label Clinical Phase III Study) also randomized refractory astrocytoma patients to AP12009 or standard chemotherapy [42].

A positive TGF-β gene-expression signature in estrogen receptor negative primary breast cancer was associated with high risk of metastasis to lung but not to the bone [43]. Novel biomarkers may be useful for assessing the clinical response to TGF-β inhibitors. 

## 4. Novel Targets to Restore Osteoblast Functions

The osteolytic lesions in bones from breast cancer results not only results from osteoclast-mediated bone resorption but inhibition of osteoblast-mediated bone formation has also a crucial role. This realization led to the development of therapeutic strategies aimed at restoring osteoblast function. 

### 4.1. DKK-1

The Wnt signaling pathway plays a key role in osteoblastogenesis. Wnt proteins bind frizzled receptor family members and in association with low density lipoprotein receptor-related protein (LRP)5/6, trigger downstream signaling via β-catenin, which includes activation of different genes involved in osteoblastogensis [44]. Elevated levels of DKK-1 were first described in the serum and bone marrow of patients with multiple myeloma [45]. The blockade of DKK-1 using neutralizing antibodies resulted in a decrease of both osteolysis and skeletal tumor growth in a severe combined immunodeficiency (SCID)-hu murine model of multiple myeloma [46]. In addition, DKK-1 antibody treatment increased in osteoblast number, serum human osteocalcin level, and trabecular bone, indicating that this antibody had bone anabolic effects [47]. A clinical trial is evaluating BHQ880 and zoledronic acid in relapsed/refractory multiple myeloma. There is pre-clinical evidence that breast cancer derived DKK-1 inhibits osteoblastogenesis [48]. Further studies are required to examine the importance of DKK-1 as a therapeutic target for breast cancer bone metastasis. 

### 4.2. Activin A

It is a member of the TGF-β superfamily of growth factors that is widely present in different cells and tissues [7]. In bone metastasis, activin A produced by tumor cells acts as a stimulator of bone of bone degradation, inhibiting osteoblast differentiation and stimulating osteoclast differentiation [50]. The circulating levels of activin A are significantly higher in patients with breast and prostate cancer than patients without bone metastasis [49]. Therefore, this cytokine may be considered as a potential target for a more selective therapeutic approach in the treatment of skeletal metastasis.

### 4.3. Endothelin-1

The endothelins are family of three small peptides: ET-1, ET-2, and ET-3 [50]. They are produced by different cells types, including breast and prostate cancer cells [50]. ET-1 is involved in the formation of osteoblasts, and it decreases osteoclast activity and motility [50]. ET-1 and ET-2 increase breast cancer cell migration and invasion *in vitro* [51]. Therefore, there is a rationale in studying the effect of endothelin antagonists in breast cancer patients. However, no clinical trials in breast cancer have been conducted so far. 

## 5. Radiotherapy and Radiopharmaceuticals

Majority of the patients get excellent palliation for localized metastatic bone pain with external beam radiotherapy. Several randomized trials have shown that a single fraction of 8 Gy is adequate for pain relief [52]. Radiopharmaceuticals are now available for the palliation of metastatic bone pain. Strontium has been shown to be as effective as wide field radiotherapy in prostate cancer [53] and because of the preferential uptake of strontium at sites of new bone formation, is probably most effective for sclerotic metastases. Samarium, which is linked to the bisphosphonates diamine tetramethylene phosphonic acid, has been evaluated in prostate and breast cancer. Samarium is also preferentially taken up at sites of bone formation, and emits both α and γ particles. The former allows imaging of the skeleton and the latter provides the therapeutic effects. Samarium is suitable for outpatient use and it has a significant effect on bone pain and analgesic consumption [54]. Further studies are indicated to compare radioisotope treatment with high-dose bisphosphonates and to determine whether the two treatment approaches complement one another. 

## 6. Conclusions

Bone metastasis is currently incurable and can be complicated by skeletal related events (SREs), which result in substantial morbidity and mortality. Bisphosphonates are currently the standard agents used for bone metastasis to reduce the frequency of the SRCs but they may have anti-tumor effects and could be useful for preventing and treating metastasis to bone and visceral sites. A more thorough understanding of the cellular and molecular mechanisms of bone metastases and bone microenvironment will help in developing novel agents. Given the complexity of the mechanisms of bone metastasis, combinations of drugs with different targets are probably needed to accomplish a successful outcome. 

This review highlights several molecular components acting at early or late stages during the development and progression of breast cancer bone metastases. These components are the new attractive targets for cancer therapeutics. They could be used in combination with bisphosphonates to efficiently block the development of skeletal lesions in women with breast cancer. 
